# Energy and Entropy Analyses of a Pilot-Scale Dual Heating HDH Desalination System

**DOI:** 10.3390/e23101282

**Published:** 2021-09-30

**Authors:** Dahiru U. Lawal, Saad Abdul Jawad, Mostafa H. Sharqawy, Mohamed A. Antar

**Affiliations:** 1 Research Center for Membranes and Water Security, King Fahd University of Petroleum and Minerals, Dhahran 31261, Saudi Arabia; dahiru.lawal@kfupm.edu.sa; 2 Mechanical Engineering Department, King Fahd University of Petroleum and Minerals, Dhahran 31261, Saudi Arabia; saadabduljawad@outlook.com; 3 School of Engineering, College of Engineering and Physical Sciences, University of Guelph, Guelph, ON N1G 2W1, Canada; melsharq@uoguelph.ca

**Keywords:** humidification-dehumidification, HDH, entropy generation, irreversibility analysis, desalination

## Abstract

This study focuses on energy and entropy analysis to theoretically investigate the performance of a pilot scale dual heated humidification-dehumidification (HDH) desalination system. Two cases of HDH systems are considered in the analysis. The first case is a dual heated (DH) cycle consisting of 1.59 kW air heater and 1.42 kW water heater with a heat rate ratio of 0.89 (CAOW-DH-I). Whereas the second case is a dual heated HDH cycle comprising of 1.59 kW air heater and 2.82 kW water heater with a heat rate ratio of 1.77 (CAOW-DH-II). As a first step, mathematical code was developed based on heat and mass transfer and entropy generation within the major components of the system. The code was validated against the experimental data obtained from a pilot scale HDH system and was found to be in a good agreement with the experimental results. Theoretical results revealed that there is an optimal mass flowrate ratio at which GOR is maximized, and entropy generation is minimized. Furthermore, the degree of irreversibility within the humidifier component is low and approaches zero, while the specific entropy generation within other components are relatively high and are of the same order of magnitude. Entropy analysis also showed that the dual heated system with heat rate ratio greater than unity is better than the one with heat rate ratio less than unity.

## 1. Introduction

Amid increasing global water scarcity, the desalinized water is widely used in municipal, agricultural, and industrial sectors. The conventional method of desalinization processes can be classified into two major categories: membrane desalination and thermal desalination. Among the membrane desalination techniques are reverse osmosis (RO) and electrodialysis (ED), while some of the conventional thermal based desalination process includes multi-stage flash (MSF) distillation, multi-effect evaporation (MEE or MED) and thermal or mechanical vapor compression systems (TVC/MVC) [1]. Although membrane distillation (MD) involves the use of hydrophobic membranes, it is still considered a thermal technique since it depends on the difference in vapor pressure due to temperature difference between the two streams on both sides of the membrane. The major thermal based desalination processes are mostly employed in large scale purification process, which is centralized and energy intensive, and they are widely driven by conventional fossil fuel resources. The use of conventional fuel resources is one of the biggest challenges facing the future of thermal desalination plants. One of the suitable thermal based desalination techniques with less dependence on conventional fossil fuel and appropriate for small–medium scale clean water production is the humidification-dehumidification (HDH) desalination process. HDH process is a vital link to the chain that seeks an energy efficient solution for potable water supplies. Over the past two decades, multiple research groups across the globe have been trying to turn HDH desalination process into an effective decentralized process by maximizing the water productivity and minimizing the energy input [2]. Furthermore, the flexibility of integrating HDH unit with renewable energy sources has poured integrative research into the subject matter, and solar and geothermal energy sources have attracted considerable attention in this aspect [3]. 

A typical HDH process consists of three primary subcomponents: a humidifier where a carrier gas (usually air) is humidified by the feedwater source, a dehumidifier where the carrier gas is dehumidified and water vapor is condensed as fresh water, and a heater where the air is heated (air heated cycle, AH) or the water is heated (water heated cycle, WH). Two conventional ways of HDH process classification are based on heating mode (i.e., AH or WH) and cycle configuration. The cycle configuration could be closed-air-open-water (CAOW) where the air (or carrier gas) is circulated inside the system and feedwater enters at high salinity and leaves as fresh water and brine, in an open loop. It could be also open-air-open-water (OAOW) where both carrier gas and water are in open loops. Furthermore, the HDH units can be categorized based on forced or natural circulation of air.

Many HDH research studies have been performed assuming either air or water heated HDH system. However, little work has been conducted for dual heated cycles where both the air and water are heated in the cycle. Studies that assumed water heated cycle found that there is an optimum water-to-air mass flow rate ratio that maximizes the water productivity at the same heat input and components efficiencies (i.e., the effectiveness of the humidifier and dehumidifier). Eslamimanesh et al. [4] modeled a CAOW-AH HDH process and concluded that, heating the air at humidifier inlet and cooling water at dehumidifier inlet increase system productivity, while increasing mass flowrate ratio in humidification column led to lower freshwater flowrate. Campos et al. [5] theoretically studied a CAOW-WH through mathematical modeling, and observed that the water productivity can be enhanced by increasing the heat absorption in the solar collector, increasing the humidifier height and decreasing seawater flowrate. The model also showed that the temperature of surrounding environment has marginal to no significant effect on the distillate flowrate. Hermosillo et al. [6] analytically investigated the performance of a CAOW-WH HDH cycle and verified their results experimentally. Results revealed that mass flow rates of air and water are the decisive factors in enhancing the water productivity. Results also reported that there is an optimum mass flowrate ratio that maximize the productivity. 

Zubair et al. [7] thermodynamically analyzed a CAOW-WH HDH cycle by using an evacuated tube solar collector as water heater. Feasibility analysis for multiple locations in Saudi Arabia was performed. With specified design parameters, the system could produce 16,430 liters of freshwater annually in Dhahran, SA. The cost of distilled water was found to be in the range of 0.032 to 0.038 $/liter. Sharqawy et al. [8] analytically investigated multiple design performance, and optimization of a CAOW water heated and modified air heated HDH desalination cycles, and presented performance charts, which can be utilized for sizing HDH systems at different operational and design conditions. Lawal et al. [9] presented a detailed mass and energy modeling of different CAOW HDH layouts integrated with a heat pump system. The performance metrics of cycle showed a maximum GOR (Gain Output Ratio) of 7.63 at humidifier and dehumidifier effectiveness of 80% and a mass flowrate ratio of 1.63. This work was escalated to a second law analysis, where entropy generated in different components of each layout was evaluated and analyzed [10]. The study concluded that exergy destroyed within the main components of the system are in the same order of magnitude. 

The concept of entropy analysis is known to be a necessary tool to quantify the thermodynamic losses of a system [11]. By applying energy and entropy analysis, Alhazmy [12] evaluated the least work needed to drive a HDH desalination system, and quantify the effective utilization of energy in the modified HDH desalination system. Mistry et al. [13] characterized many HDH desalination cycles and identified ways to improve the HDH cycles and its components through entropy analysis. A sink and source model has also been employed to perform second law analysis on an HDH system [14]. Based on the survey, it can be concluded that entropy generation is a vital tool in assessing the actual performance of a system by evaluating losses associated with major components of a system. Therefore, the present work is aimed at modeling a pilot scale dual (air and water) heated humidification-dehumidification system. Mathematical model based on first law of thermodynamics is developed and validated against the experimental data of a pilot scale dual heated HDH unit. Thereafter, second law analysis is performed based on the developed theoretical model to assess the entropy generation on the major components of the system. 

## 2. System Description

**Figure 1:**
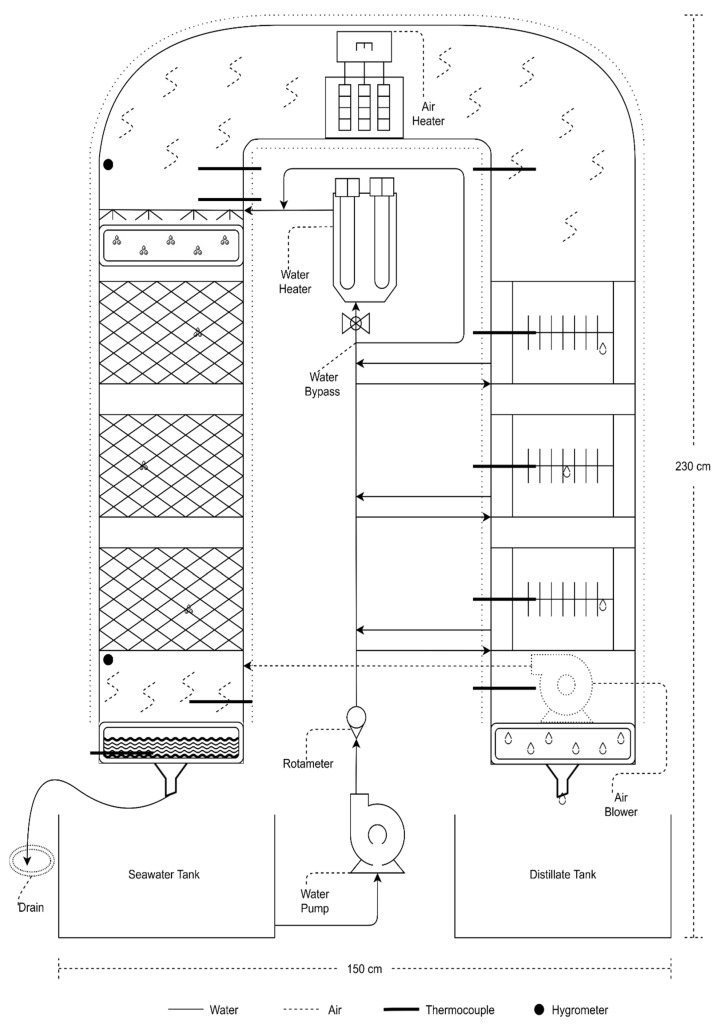
Schematic layout of Experimental Testing Rig.

The system under investigation is a pilot scale CAOW HDH system with dual heating of air and water streams. The system is schematically illustrated in Figure 1. The pilot scale unit is mounted on a rigid frame made of iron-steel and it is of 2.3 m height and 1.5 m width. In this system, the humidifier and dehumidifier columns have 30 cm × 30 cm cross section and are made from galvanized steel sheets. The feedwater enters a set of three fin-and-tube dehumidifiers in-series at an ambient temperature where it ascends through the dehumidifier tubes and takes in the heat of condensation during its upward trail. Meanwhile, outgoing heated air in the dehumidifier interacts with the incoming water indirectly via fins to discharge the moisture it is holding, as freshwater. Preheated feedwater from the dehumidifier enters a water heating unit where its temperature gets elevated further. The heated water is then sprayed at the top of the humidifier using nozzles. In the humidifier, direct heat and mass exchange take place between the two interacting streams. Air gets heated and humidified and then advances into the air heating unit, where sensible heat is added to the preheated moist air stream. Sequentially, air then circulates in a closed loop and re-enters the humidifier after losing its moisture content in the dehumidifier. 

The humidifier column (left column in Figure 1 schematic) is a packed-bed direct heat and mass exchanger of 1.7 m height. Spray nozzles are affixed at the top of the humidifier column to disperse pre-heated seawater coming out from the dehumidifier over the packing material, which increases direct heat and mass exchange between the two interacting fluid streams. Underneath the packing material is the brine collector which discharges unevaporated saline water out from the humidifier. The dehumidifier column (right column in Figure 1 schematic) consists of 3 finned tube heat exchangers of equal size and cooling capacity. The main purpose of finned tube exchangers is to condense the freshwater out from hot-humid air coming from the humidifier. Other than cooling the moist air down, these indirect heat and mass exchangers also recover the heat from hot air by heating the incoming seawater. At the bottom of the dehumidifier is the freshwater collector or distillate tank. The heating units are incorporated between the humidifier and dehumidifier. This desalination system is capable of heating air and/or water in separate electric heaters. Air stream can be heated after the humidifier with variable air heaters up to 5.5 kW heating capacity. Whereas the water heaters are installed before the humidification process using variable electric heaters up to 3 kW capacity. 

The system is equipped with 0.6 hp water pump (Calpeda) and 0.37 kW variable speed air blower for water and air circulation respectively. In addition, the system has many instruments to measure different experimental parameters. An in-line flow type rotameter (Omega FL45100) and turbine flowmeter (Omega FTB4605) are used to monitor and measure the water flow rate flowing into the dehumidifier. Rugged pipe K-type thermocouple probes are used to measure water temperatures in the pipes. Air temperatures across the circuit are also measured with K-type thermocouple probes housed inside a metallic stainless-steel tube. Duct type hygrometers or relative humidity sensor is used to record relative humidity at the inlet and exit of the humidifier (Omega HX94V by Omega Engineering Inc. 800 Connecticut Ave. Suite 5N01, Norwalk, CT 06854, USA). Analogue voltmeter and ammeter devices are used to read the voltage and current being supplied to the rig and consequently the power consumed by each electric component of the system. A data logger (Fluke 2686A DAQ by Fluke Corporation, 6920 Seaway Blvd Everett, WA 98203 United States) is used to receive the voltage signals from different recorders including thermocouples, relative humidity sensors and flow meters. 

## 3. Mathematical Model

**Figure 2:**
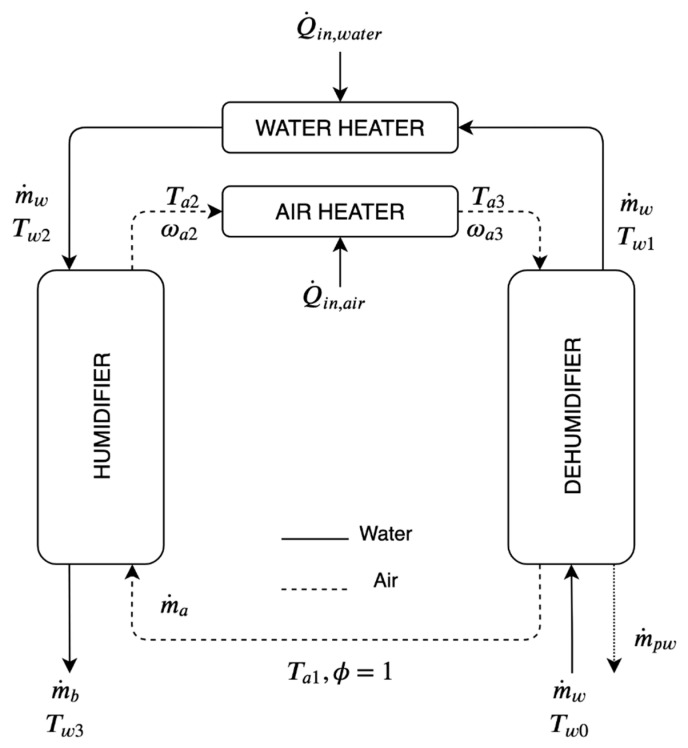
Schematic Diagram of a Dual Heated HDH Cycle.

Analytical model for closed air-open water (CAOW) dual heated (DH) (air and water) HDH desalination cycle is presented in this section. Energy and mass balances are applied to substantiate the heat and mass exchange processes. Figure 2 represents a functioning layout of a CAOW-DH-HDH cycle. The developed system of equations has been solved using the Engineering Equation Solver (EES) software. In model development, the following assumptions are made:

- The processes involved in the cycle operate at steady state conditions.
- Negligible heat loss occurs through the subcomponents, in the cycle.
- Power needed to operate the pump and fan is minimal in comparison to the input required by heating unit.
- Produced freshwater is expected to leave the dehumidifier at a mean temperature between the temperature of humid air at the entrance and temperature of air at the exit.
- Relative humidity of air that leaves both the system subcomponents is assumed to be >90%. Since, air gets heated and humidified simultaneously in an evaporator. Similarly, air loses heat and moisture as it flows through the dehumidifier.

### Governing Equations

Mass balance and energy balance in humidifier of a DH-HDH cycle are expressed in the respective equations below;

(1) $${\dot{m}}_{w}={\dot{m}}_{b}+{\dot{m}}_{pw}$$

(2) $${\dot{m}}_{a}h_{a1}+{\dot{m}}_{w}h_{w2}={\dot{m}}_{b}h_{w3}+{\dot{m}}_{a}h_{a2}$$

Entropy balance of the humidifier is given as,

(3) $${\dot{S}}_{gen,hum.}={\dot{m}}_{a}s_{a2}+{\dot{m}}_{b}s_{w3}-{\dot{m}}_{a}s_{a1}-{\dot{m}}_{w}s_{w2}\geq 0$$

Effectiveness of humidifier is defined as [15];

(4) $$\varepsilon_{hum.}=max\langle \frac{{\dot{m}}_{w}h_{w2}-{\dot{m}}_{b}h_{w3}}{{\dot{m}}_{w}h_{w2}-{\dot{m}}_{b}h_{w3,ideal}},\frac{\left(h_{a2}-h_{a1}\right)}{\left(h_{a2,ideal}-h_{a1}\right)}\rangle$$

Similarly, mass and energy balance equations for dehumidifier are formulated below.

(5) $${\dot{m}}_{pw}={\dot{m}}_{a}\left(\omega_{a3}-\omega_{a1}\right)$$

(6) $${\dot{m}}_{w}h_{hw0}+{\dot{m}}_{a}h_{a3}={\dot{m}}_{w}h_{w1}+{\dot{m}}_{a}h_{a1}+{\dot{m}}_{pw}h_{pw}$$

Entropy balance of the dehumidifier is stated as,

(7) $${\dot{S}}_{gen,dehum.}={\dot{m}}_{w}s_{w1}+{\dot{m}}_{a}s_{a1}+{\dot{m}}_{pw}s_{pw}-{\dot{m}}_{w}s_{w0}-{\dot{m}}_{a}s_{a3}\geq 0$$

Effectiveness of dehumidifier is expressed as [15];

(8) $$\varepsilon_{dehum.}=max\langle \frac{h_{w1}-h_{w0}}{h_{w1,ideal}-h_{w0}},\frac{h_{a3}-h_{a1}}{h_{a3}-h_{a1,ideal}}\rangle$$

Energy and entropy balance for the air heater and water heater is presented here.

(9) $${\dot{Q}}_{in,air}={\dot{m}}_{a}\left(h_{a3}-h_{a2}\right)$$

(10) $${\dot{Q}}_{in,water}={\dot{m}}_{w}\left(h_{w2}-h_{w1}\right)$$

(11) $${\dot{S}}_{gen,heater,air}={\dot{m}}_{a}\left(s_{a3}-s_{a2}\right)-\frac{{\dot{Q}}_{in,air}}{T_{0}}\geq 0$$

(12) $${\dot{S}}_{gen,heater,water}={\dot{m}}_{w}\left(s_{w2}-s_{w1}\right)-\frac{{\dot{Q}}_{in,water}}{T_{0}}\geq 0$$

Heat rate ratio is defined as ratio between total energy rate inputs to water over air as;

(13) $$Q_{r}=\frac{{\dot{Q}}_{in,water}}{{\dot{Q}}_{in,air}}$$

The performance of the cycle is measured by the Gained Output Ratio (GOR), which is computed as the enthalpy of evaporation multiplied by the distillate water flow rate to the total thermal energy input. It outlines the efficacy of system in producing fresh water and gives an estimate about the reinstated heat recovery, in a desalination cycle.

(14) $$GOR=\frac{{\dot{m}}_{pw}\times h_{fg}}{{\dot{Q}}_{in,air}+{\dot{Q}}_{in,water}}$$

The Mass Flow Rate Ratio (MR) is another important parameter in the cycle, which is calculated by dividing the mass flowrate of inlet saline water to the air being circulated in the cycle. At an optimum value of MR, the GOR is maximum.

(15) $$MR=\frac{{\dot{m}}_{w}}{{\dot{m}}_{a}}$$

For fixed component effectiveness and mass flow rate ratio, the dual heated humidification-dehumidification desalination cycle in semi-open air-open water configuration has shown better system performance when heat rate ratio is equal to or greater than unity [16].

## 4. Results and Discussion

The validation of the mathematical model against the experimental findings and the results of the theoretical entropy analysis are discussed in this section. Specifically, the observed performance indicators of the experimental data are validated, by comparing them with numerical solution from the mathematical models. 

### 4.1. Model Validation with Experimental Data

**Figure 3:**
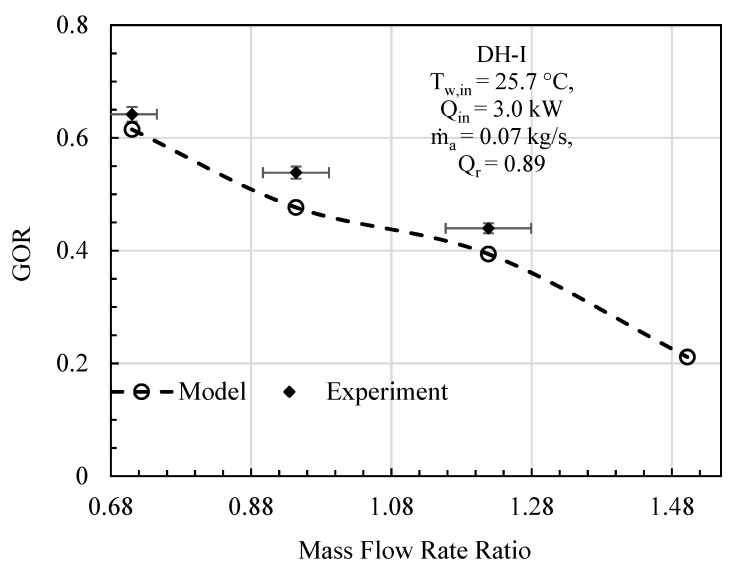
DH-I Cycle Theo & Exp: Mass flow rate ratio vs. gained output ratio.

**Figure 4:**
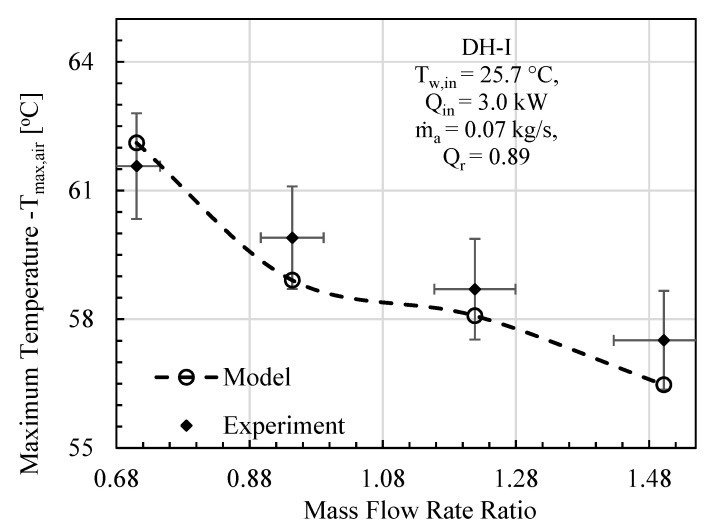
DH-I Cycle Theo & Exp: Mass flow rate ratio vs. maximum cycle temperature.

**Figure 5:**
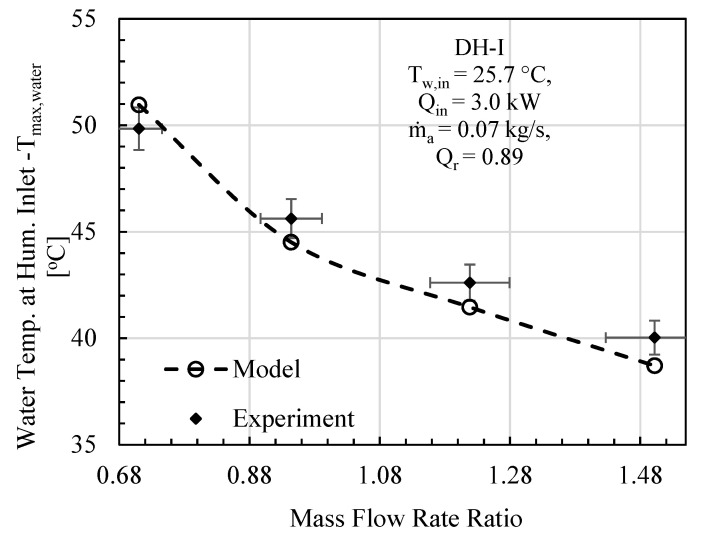
DH-I Cycle Theo & Exp: Mass flow rate ratio vs. water temp. at hum. inlet.

**Figure 6:**
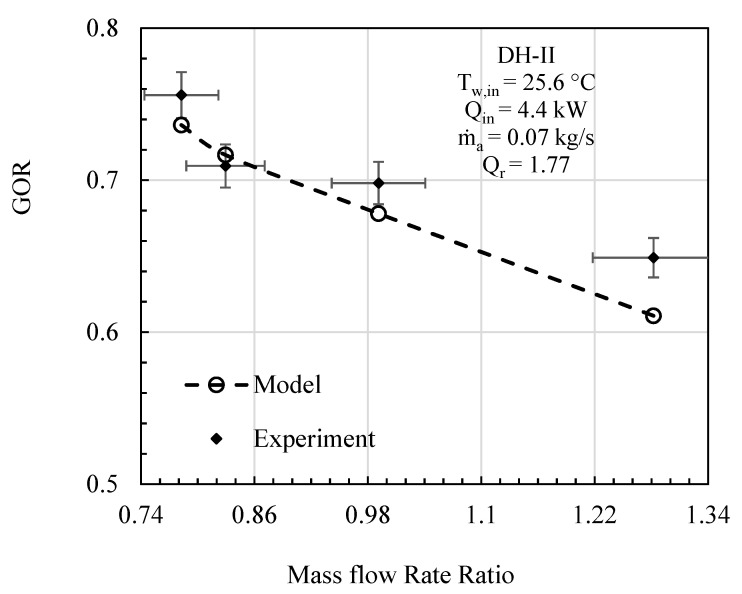
DH-II Cycle Theo & Exp: Mass flow rate ratio vs. gained output ratio.

**Figure 7:**
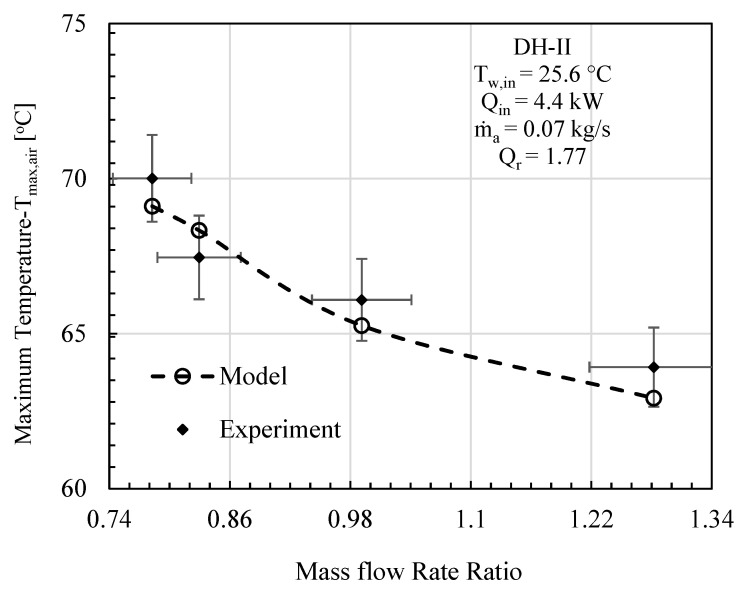
DH-II Cycle Theo & Exp: Mass flow rate ratio vs. maximum cycle temperature.

**Figure 8:**
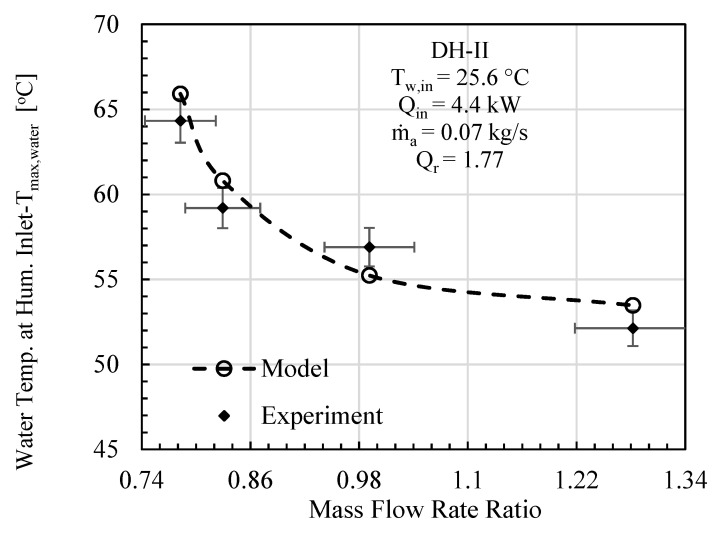
DH-II Cycle Theo & Exp: Mass flow rate ratio vs. water temp. at hum. inlet.

In this comparative study, mass flowrate ratio, energy input, initial water temperature, effectiveness, and relative humidity are given as input to the mathematical model, and the specific experimental test conditions are provided on each figure. The comparative analysis was performed based on gained output ratio and stream temperatures across the cycle for DH-I (Figure 3, Figure 4 and Figure 5) and DH-II (Figure 6, Figure 7 and Figure 8). DH-I means dual heated cycle consisting of 1.59 kW air heater and 1.42 kW water heater with a heat rate ratio (*Q_r_*) of 0.89. Whereas DH-II represents the case of dual heated cycle comprising 1.59 kW air heater and 2.82 kW water heater with a heat rate ratio (*Q_r_*) of 1.77. Equation (13) provides the expression for the heat rate ratio.

For DH-I cycle, the comparison between experimental and theoretical results in terms of GOR is designated in Figure 3. It can be deduced from the analysis that overall variation trend of experimental GOR with MR remained the same with the GOR obtained from the mathematical model. The maximum percentage deviation of experimental GOR from theoretical GOR is found to be about 11%, which is recorded at mass flowrate ratio of 0.94. The main contributing factor to this disparity may be attributed to the product water mass flowrate ${\dot{m}}_{\mathrm{pw}}$. This is anticipated because, after achieving the steady state, the feedwater coming from storage tank observes slight temperature variations, including minor fluctuation in ${\dot{m}}_{w}$ that is controlled via a ball valve. However, when it comes to computation, the reported data has been averaged to minimize noise in the recorded data. Likewise, the theoretical model is processing these averaged values of recorded parameters that tend to give slight differences in productivity. To further strengthen the case, experimental top cycle air temperature (*T_max,air_*) is compared with mathematically computed maximum cycle temperature of the dual heated HDH desalination cycle and demonstrated in Figure 4. It is evident from the figure that the analytical and experimental temperatures agreed well with each other, with a maximum difference of 1.04 °C, at mass flowrate ratio of 1.5. Similarly, the comparison in terms of system maximum water temperature (after exiting the heating unit and before entering humidifier) is also illustrated in Figure 5. A good agreement between observed and computed values can be witnessed as well. The largest difference between experimental and theoretical values was found to be 1.32 °C and was recorded at MR of 1.5. 

By increasing the heat rate ratio Equation (14), the overall performance of the dual heated HDH system was observed to improve. Figure 6 illustrates the comparison between the analytical and experimental performance of dual heated system with total heat input of 4.4 kW (heat rate ratio (*Q_r_*) = 1.77). A maximum percentage error of 5.89% was noticed between theoretical and experimental results at MR of 1.28. The contrast between the model and experimental data in terms of maximum cycle air temperature (*T_max,air_*) is given in Figure 7. The highest temperature difference between mathematical and experimental values was found to be 1.56 °C, which was recorded at mass flowrate ratio of 1.28. Similarly, maximum cycle water temperature is presented under the specified initial conditions of DH-II system in Figure 8. It has been found that maximum disparity between the experimental and analytical *T_max,water_* was 2.93 °C, at mass flowrate ratio of 0.99. 

In conclusion, the model results were found to agreed well with the experimental findings, with a maximum percentage deviation of about ±11.48%. Therefore, the presented mathematical model guaranteed good prediction capability and can be utilized for further analysis (entropy analysis).

Based on the considered ranges of experimental conditions, a maximum GOR of 0.756 was obtained experimentally. The obtained GOR is quite low when compared to most of the reported GOR in the literature as tabulated in Table 1. However, the current GOR is also higher than some other reported GOR in the literature. For example, the present dual heated HDH desalination unit registered an improved GOR against the experimental work of Deniz and Çınar [17], Khalil et al. [18] and Zubair et al. [19], whose system are dual heated, air heated, and water heated HDH desalination systems, respectively. It is worth noting that most of the systems with higher GOR are lab-scale system where most of the operating conditions are well controlled with minimum heat lost by the system.

### 4.2. Entropy Analysis

**Figure 9:**
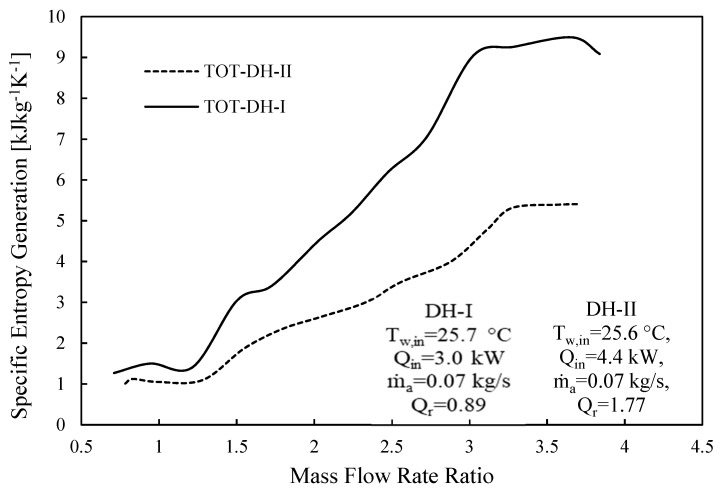
Specific entropy generation vs. MR for CAOW-DH-I and CAOW-DH-II.

This section discusses entropy analysis based on the developed mathematical codes. Figure 9 illustrates the specific entropy generation as a function of mass flowrate ratio for the two cases under considerations (DH-I and DH-II). For DH-I and DH-II, *Q_r_* is 0.89 and 1.77, respectively. The specific entropy generation represents the cycle’s total entropy generation per freshwater mass flowrate. 

It can be noticed that the minimum specific entropy generation for CAOW-DH-I and CAOW-DH-II corresponds to the highest gained output ratio as presented in Figure 3 and Figure 6, respectively. This means that for each case, optimal condition exists at which GOR is maximized, and entropy generation is minimized. It can also be observed that the entropy generated in case of DH-I is higher than that generated in DH-II. This support the reason why the GOR in DH-II system is higher than the GOR of DH-I case. This result also shows that dual heated HDH system with heat rate ratio higher than unity is better than dual heated HDH system with heat rate ratio less than unity.

**Figure 10:**
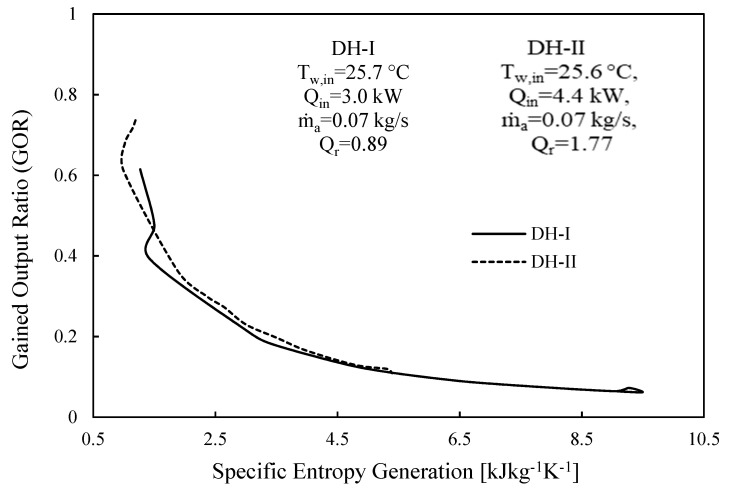
GOR against the specific entropy generation for CAOW-DH-I and CAOW-DH-II.

Figure 10 demonstrates the case of GOR as a function of specific entropy generation for CAOW-DH-I and CAOW-DH-II. It can be observed that, as the specific degree of irreversibility within the cycle reduces, the gained output ratio of the system increases. This is expected, since it falls within the fact that the performance of a thermal system improves with reduction in the degree of irreversibility.

**Figure 11:**
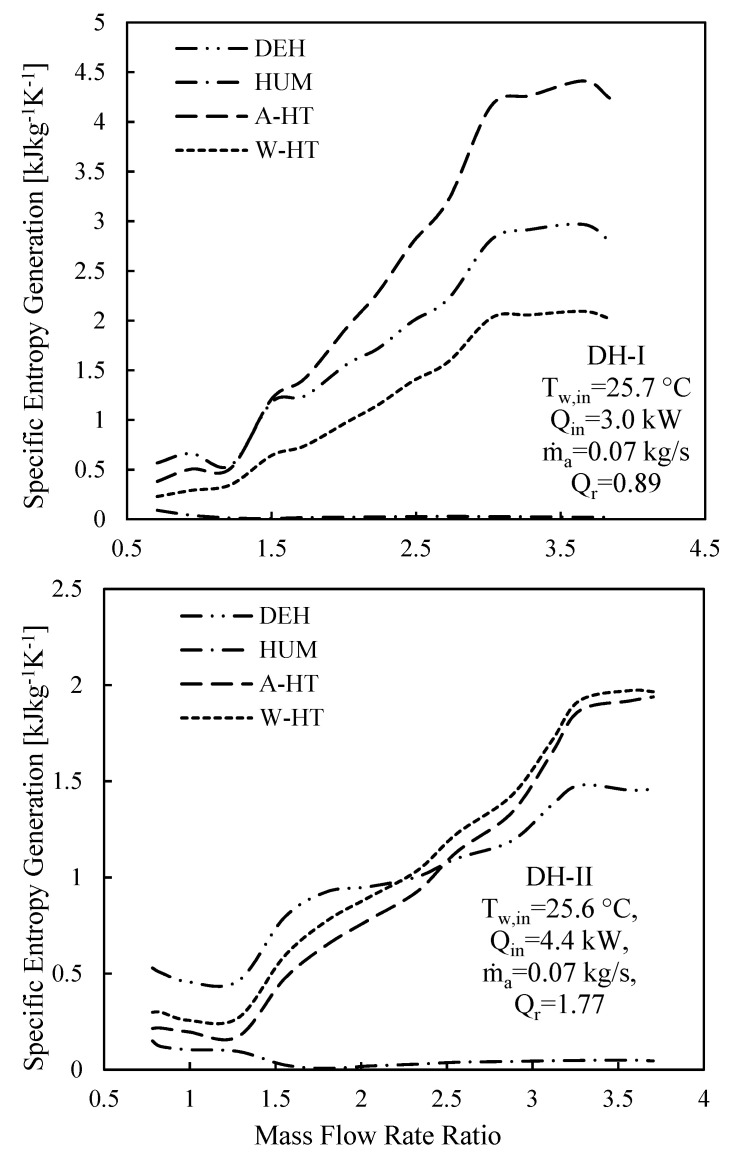
Specific entropy generation in each component of CAOW-DH-I and CAOW-DH-II.

The entropy generation within each component of CAOW-DH-I and CAOW-DH-II per pure water mass flowrate is depicted in Figure 11. The specific entropy generation within each component of each cycle is plotted as a function of the MR. With exception of humidifier, the degree of irreversibilities within the dehumidifier, air heater, and water heater are quite high and are of the same order of magnitude. This signifies that caution needs to be observed when designing these components (dehumidifier, air heater, and water heater). Furthermore, the irreversibilities within the humidifier is relatively low and approaches zero. This implies that humidifier cannot be further improved to have a greater effectiveness. In fact, experimental data showed that the effectiveness of the humidifier is in the range of 72–100%. Therefore, to improve the overall performance of the system, efforts should be channeled towards improving the effectiveness of the dehumidifier rather than both components in this case. 

## 5. Conclusions

- Results of the theoretical model have been validated against the experimental outcomes, and the model was found to be in a good agreement with the experimental findings. It has been found that the maximum overall percentage deviation from the model is within 11.5% of the experimental data.
- The concept of heat rate ratio *Q_r_* is introduced, which signifies the effect of total energy distribution between water and air streams, in dual heated systems. It is deduced that the system performs better when heat rate ratio is greater than one or when more energy is supplied to the water stream.
- There exists an optimal mass flowrate ratio at which GOR is maximized, and entropy generation is minimized.
- The specific entropy generation within the humidifier is low and approaches zero, while the degree of irreversibilities within other components are quite high and are of the same order of magnitude.
- Both energy (GOR) and entropy analysis revealed that dual heated HDH system with heat rate ratio greater than unity is better that dual heated HDH system with heat rate ratio lower than unity.

## Figures and Tables

**Table 1:** Comparison of GOR between the present system and other HDH systems.

Energy Source	GOR	Ref
Solar Energy (Experiment)	2.2	[20]
Solar Energy (Experiment)	2.1	[21]
Solar Energy (Experiment)	0.3154	[17]
Solar Energy (Experiment)	0.53	[18]
Electric Heater (Experiment)	0.42	[19]
Electric Heater (Experiment)	2.7	[22]
Electric Heater (Experiment)	1.3	[23]
Heat Pump (Experiment)	2.08	[24]
Heat Pump (Theory)	2.532	[25]
Heat Pump (Theory)	0.76	[26]
Heat Pump (Experiment)	2.05	[27]
Heat Pump (Theory)	2.476	[28]
Heat Pump (Theory)	3.91	[29]
Electric Heater (Experiment)	0.756	Present

## Data Availability

Not Applicable.

## Nomenclature

*Symbols*	
*h*	specific enthalpy (kJ/kg)
*h_fg_*	latent heat of vaporization (kJ/kg)
*GOR*	gain output ratio
*MR*	mass flowrate ratio
*ṁ_w_*	feedwater mass flowrate (kg/s)
*ṁ_pw_*	freshwater mass flowrate (kg/s)
*ṁ_b_*	brine mass flowrate (kg/s)
*ṁ_a_*	dry air mass flowrate (kg/s)
$\dot{Q}$ *_in_*	rate of heat input (kW)
*Q_r_*	heat rate input ratio [$\dot{Q}$*_in,water_*/$\dot{Q}$*_in,air_*] (-)
*s*	specific entropy (kJ/kg.K)
$\dot{S}$ *_gen_*	entropy generation rate (kW/K)
$\dot{s}$ *_gen_*	specific entropy generation (kJ/kg.K)
*T*	temperature (C)
*ε*	effectiveness (-)
*ω*	humidity ratio (kg_*w*_/kg_*a*_)
*Subscripts*	
*a*	air
*d*	dehumidifier
*h*	humidifier
*ht*	heater
*Hum./Deh.*	humidifier/dehumidifier
*w*	water

## References

[1] Lawal D.U., Qasem N.A.A. (2020). Humidification-dehumidification desalination systems driven by thermal-based renewable and low-grade energy sources: A critical review. Renew. Sustain. Energy Rev..

[2] Narayan G.P., Lienhard V.J.H., Kucera I.J. (2014). Humidification Dehumidification Desalination. Desalination: Water from Water.

[3] Bourouni K., Martin R., Tadrist L., Chaibi M.T. (2002). Heat transfer and evaporation in geothermal desalination units. Appl. Energy.

[4] Eslamimanesh A., Hatamipour M.S., Eslamimanesh A.H.M. (2009). Mathematical modeling of a direct contact humidification-dehumidification desalination process. Desalination.

[5] De Oliveira Campos B.L., da Costa A.O.S., da Costa Junior E.F. (2017). Mathematical modeling and sensibility analysis of a solar humidification-dehumidification desalination system considering saturated air. Sol. Energy.

[6] Hermosillo J.J., Arancibia-Bulnes C.A., Estrada C.A. (2012). Water desalination by air humidification: Mathematical model and experimental study. Sol. Energy.

[7] Zubair M.I., Al-Sulaiman F.A., Antar M.A., Al-Dini S.A., Ibrahim N.I. (2016). Performance and cost assessment of solar driven humidification dehumidification desalination system. Energy Convers. Manag..

[8] Sharqawy M.H., Antar M.A., Zubair S.M., Elbashir A.M. (2014). Optimum thermal design of humidification dehumidification desalination systems. Desalination.

[9] Lawal D., Antar M., Khalifa A., Zubair S., Al-Sulaiman F. (2018). Humidification-dehumidification desalination system operated by a heat pump. Energy Convers. Manag..

[10] Lawal D.U., Zubair S.M., Antar M.A. (2018). Exergo-economic analysis of humidification-dehumidification (HDH) desalination systems driven by heat pump (HP). Desalination.

[11] Muthusamy C., Srithar K. (2015). Energy and exergy analysis for a humidification-dehumidification desalination system integrated with multiple inserts. Desalination.

[12] Alhazmy M.M. (2007). Minimum work requirement for water production in humidification-dehumidification desalination cycle. Desalination.

[13] Mistry K.H., Lienhard J.H., Zubair S.M. (2010). Effect of entropy generation on the performance of humidification-dehumidification desalination cycles. Int J. Therm. Sci..

[14] Ashrafizadeh S.A., Amidpour M. (2012). Exergy analysis of humidification-dehumidification desalination systems using driving forces concept. Desalination.

[15] Lawal D.U., Antar M.A., Khalifa A.E. (2021). Integration of a MSF Desalination System with a HDH System for Brine Recovery. Sustainability.

[16] Mahdizade E.Z., Ameri M. (2018). Thermodynamic investigation of a semi-open air, humidification dehumidification desalination system using air and water heaters. Desalination.

[17] Deniz E., Çınar S. (2016). Energy, exergy, economic and environmental (4e) analysis of a solar desalination system with humidification-dehumidification. Energy Convers. Manag..

[18] Khalil A., El-Agouz S.A., El-Samadony Y.A.F., Abdo A. (2015). Solar water desalination using an air bubble column humidifier. Desalination.

[19] Zubair S.M., Antar M.A., Elmutasim S.M., Lawal D.U. (2018). Performance evaluation of humidification-dehumidification (HDH) desalination systems with and without heat recovery options: An experimental and theoretical investigation. Desalination.

[20] Hamed M.H., Kabeel A.E., Omara Z.M., Sharshir S.W. (2015). Mathematical and experimental investigation of a solar humidification–dehumidification desalination unit. Desalination.

[21] Wu G., Zheng H., Ma X., Kutlu C., Su Y. (2017). Experimental investigation of a multi-stage humidification-dehumidification desalination system heated directly by a cylindrical Fresnel lens solar concentrator. Energy Convers. Manag..

[22] Lawal D.U., Antar M.A., Aburub A., Aliyu M. (2018). Performance assessment of a cross-flow packed-bed humidification-dehumidification (HDH) desalination system—The effect of mass extraction. Desalin. Water Treat..

[23] Aburub A., Aliyu M., Lawal D.U., Antar M. (2017). Experimental investigations of a cross-flow humidification dehumidification desalination system. Int. Water Technol. J. IWTJ.

[24] Shafii M.B., Jafargholi H., Faegh M. (2018). Experimental investigation of heat recovery in a humidification-dehumidification desalination system via a heat pump. Desalination.

[25] Zhang Y., Zhang H., Zheng W., You S., Wang Y. (2019). Numerical investigation of a humidification-dehumidification desalination system driven by heat pump. Energy Convers. Manag..

[26] Dehghani S., Date A., Akbarzadeh A. (2018). Performance analysis of a heat pump driven humidification-dehumidification desalination system. Desalination.

[27] Zhang Y., Zhu C., Zhang H., Zheng W., You S., Zhen Y. (2018). Experimental study of a humidification-dehumidification desalination system with heat pump unit. Desalination.

[28] Faegh M., Shafii M.B. (2019). Performance evaluation of a novel compact humidification-dehumidification desalination system coupled with a heat pump for design and off-design conditions. Energy Convers. Manag..

[29] Lawal D.U., Antar M.A., Khalifa A., Zubair S.M. (2020). Heat pump operated humidification-dehumidification desalination system with option of energy recovery. Sep. Sci. Technol..

